# miR-590 promotes cell proliferation and invasion in T-cell acute lymphoblastic leukaemia by inhibiting RB1

**DOI:** 10.18632/oncotarget.8414

**Published:** 2016-03-28

**Authors:** Mei-hua Miao, Xue-qiang Ji, Hao Zhang, Jun Xu, Hong Zhu, Xue-jun Shao

**Affiliations:** ^1^ Department of Clinical Laboratory Diagnosis, Children's Hospital of Soochow University, Suzhou, China; ^2^ Department of Hematology, Affiliated Hospital of Jining Medical University, Jining, China

**Keywords:** miR-590, RB1, T-ALL, cancer

## Abstract

MicroRNAs play important roles in the pathogenesis of cancers by inhibiting gene expression at posttranscriptional level. Here, we identified that miR-590 and its predicted target gene RB1 are differentially expressed in T-cell acute lymphoblastic leukaemia (T-ALL). The correlation between miR-590 and RB1 was further confirmed in 395 T-ALL patients. In T-ALL cell lines, miR-590 promoted the cell proliferation by increasing G1/S transition. Moreover, migration and invasion assay showed that miR-590 promotes the migration and invasion of T-ALL cells by increasing E-cadherin and inhibiting MMP-9. Luciferase assays confirmed that miR-590 directly binds to the 3′untranslated region of RB1, and western blotting showed that miR-590 suppresses the expression of RB1 at the protein levels. This study indicated that miR-590 inhibits RB1 and promotes proliferation and invasion of T-ALL cells. Thus, miR-590 may represent a potential therapeutic target for T-ALL intervention.

## INTRODUCTION

Acute lymphoblastic leukemia (ALL) is the most common leukemia in pediatrics. About 80% ALL cases occur in children. It's a hematologic malignancy arising from the hematopoietic precursors of lymphoid. T-cell acute lymphoblastic leukemia (T-ALL) is the ALL transformed from the developing thymocytes. It's the result of cooperative genetic lesions. These genetic aberrations affect various biological processes, e.g. self-renewal, proliferation and survival, as well as the differentiation of precursor T cells [1].

The discovery of miRNAs opened a new generation of understanding the carcinogenesis, especially leukemogenesis [2]. miRNAs are small, non-coding RNAs that negatively regulate the gene expression by translational repression or mRNA degradation [3]. Deregulated miRNA would disrupt the hematopoietic system and arise leukemia. Many miRNAs, e.g. miR-2909 [4], miRNA-193b-3p [5], miRNA-128-3p [6] and miRNA-100/99a [7], are involved in the carcinogenesis of T-ALL.

In this study, we analyzed the miRNA and mRNA expression changes in T-ALL and identified miR-590 as a new potential T-ALL-related miRNAs with a predicted and target gene RB1. The correlation between miR-590 and RB1 was further confirmed in 395 T-ALL patients. Wet experiments in T-ALL cell lines revealed that miR-590 directly binds to the 3′untranslated region of RB1, and suppresses the expression of RB1. Moreover, inhibition of miR-590 expression would reduce proliferation and invasion of T-ALL cell lines.

## RESULTS

### miR-590 is predicted to be the key regulator of RB1 in T-ALL

**Figure 1:**
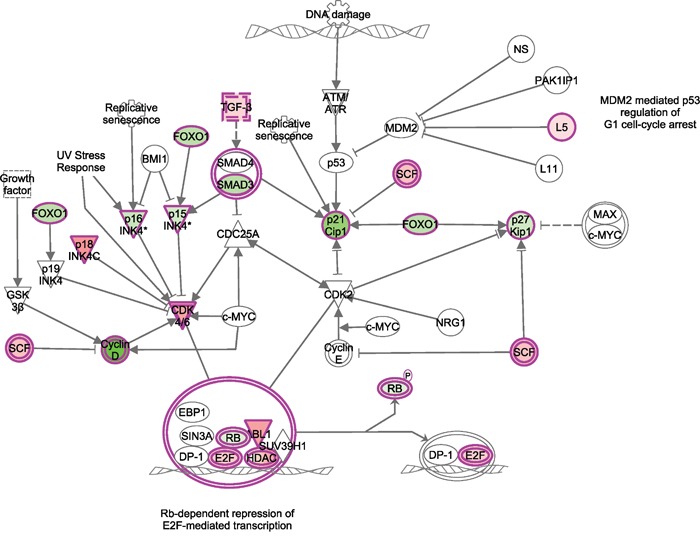
Differentially expressed miRNAs and corresponding target genes in T-ALL are enriched in cell cycle pathway Red means this molecule was up-regulated in ALL, while green means down-regulated.

**Figure 2:**
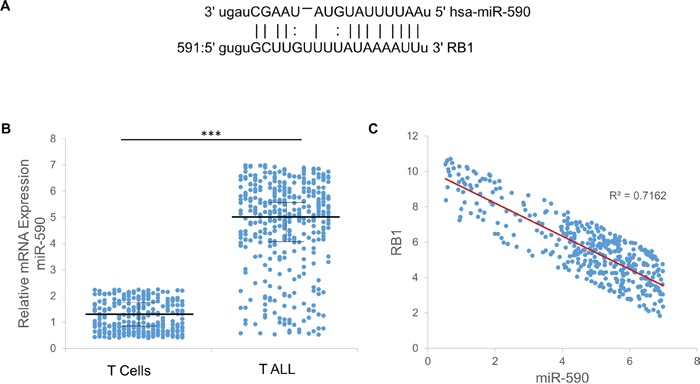
Expression of miR-590 and RB1 in T-ALL patients **A.** The complementarity between miR-590 and RB1. **B.** The expression of miR-590 in the T-ALL patients and age-matched controls. **C.** The inverse correlation between miR-590 and RB1 in the T- ALL patients was determined by Pearson's correlation coefficient (R = −0.7216, p < 0.001). *p<0.05, **p<0.01, and ***p<0.001.

The comparison of the transcriptome profiles between T-ALL samples and normal healthy controls identified that 135 miRNAs and 357 genes were differentially expressed in T-ALL. Pathway enrichment analysis indicated that differentially expressed genes of T-ALL are significantly enriched (P. value < 0.001) in Cell cycle pathway (Figure 1). In this pathway, Rb1 drew our specific attention. It plays the central role among all the differentially expressed genes in this pathway (Figure 1). Interestingly, miRNA targets prediction analysis indicated that there is a potential RB1 regulatory miRNA, miR-590, in the identified 135 differentially expressed genes of T-ALL (Figure 2A). Therefore, we selected miR-590 and RB1 for further analysis in the human blood samples.

### The expressions of miR-590 and RB1 are highly correlated in T-ALL

The blood samples were obtained from 395 patients with T-ALL and 316 age-matched healthy controls were collected between November 2013 and June 2015 at the Children's Hospital of Soochow University (Suzhou, China) and had not received local or systemic treatment (Table 1). The RT-PCR analysis indicated that expression of miR-590 is higher in T-ALL patients comparing with age-matched healthy controls (Figure 2B). Moreover, the expression of RB1 is highly correlated (R^2^=0.7162) with miR-590 in T-ALL patients (Figure 2C). It leads to the hypothesis that miR-590 might play roles in T-ALL by regulating RB1 expression. Therefore, more investigations were performed to study the regulatory interaction between miR-590 and RB1.

**Table 1:** Clinical and immunophenotypic features of T-ALL patients and normal controls

Characteristics	Patient (n=395)	Controls (n=316)	*P*-value
Age^1^, years	12.8 (1 – 26)	13.1 (1 – 29)	0.79
Gender (M/F)	395(219/176)	316 (196/120)	0.12
Hemoglobin^1^ (g/l)	10.4 (3.6 – 15.6)	166.4 (104.6 – 195)	<0.001***
WBC^1^ count × 10^9^/l	194 (4.6 – 314)	16.3 (4.5 – 20)	<0.001***
Platelets^1^ × 10^9^/l	109.4 (9 – 652)	148.8 (100 – 285)	0.03*
Pre thymic (%)	115/395 (29)	54/316 (17)	0.21
Thymic (%)	114/395 (29)	133/316 (42)	0.51
Mature T (%)	166/395 (33)	129/316 (33)	0.37

WBC: white blood cell;

* Significant *P*-value.

1 Values represent mean (range).

### MiR-590 targets and negatively regulates RB1 in T-ALL cells

**Figure 3:**
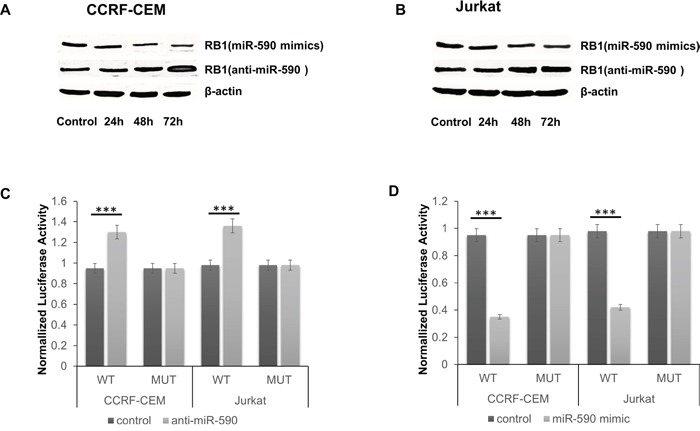
miR-590 targets RB1 in T-ALL cells **A.** and **B.** Protein of RB1 significantly changed with time after transfection with miR-590 mimics, anti-miR-590 or inactive control in CCRF-CEM and Jurkat cells; **C.** The analysis of the relative luciferase activities of RB1-WT, RB1-MUT in CCRF-CEM and Jurkat cells after transfection with anti-miR-590; **D.** The analysis of the relative luciferase activities of RB1-WT, RB1-MUT in CCRF-CEM and Jurkat cells after transfection with miR-590 mimics; *p<0.05, **p<0.01, and ***p<0.001.

As predicted by miRanda [11] and TargetScan [12], there is complementarity between miR-590 and the 3′ UTR of RB1 (Figure 2A). miR-590 mimics reduced the protein levels of RB1 in CCRF-CEM and Jurkat T-ALL cells, while miR-590 inhibitors increased the protein levels of RB1 (Figure 3A and 3B). The effect of miR-590 on the translation of RB1 mRNA into protein was then determined by luciferase reporter assay. miR-590 mimics reduced the luciferase activity of the reporter gene with the wild type but not with the mutant RB1 3′UTR construct, while the miR-590 inhibitors increased the luciferase activity of the reporter gene with the wild type but not with the mutant RB1 3′UTR construct (Figure 3B and 3C). These evidences indicate that miR-590 directly targets the 3′UTR region of RB1 and negatively regulates RB1 expression. Since RB1 is a negative regulator of the cell cycle [13], cell proliferation assays were performed to investigate the role of miR-590 in T-ALL.

### miR-590 promotes T-ALL cell proliferation by increasing G1/S transition

**Figure 4:**
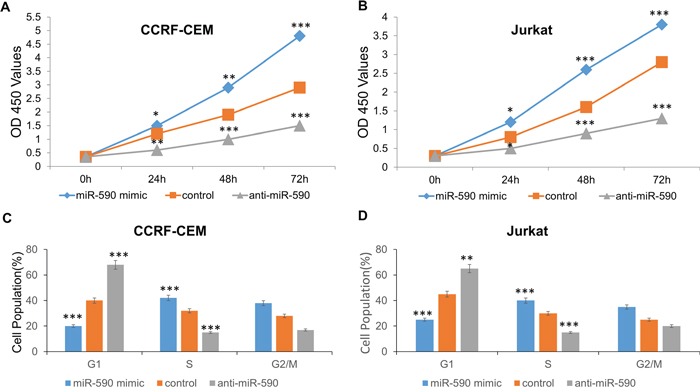
miR-590 regulates T-ALL cell proliferation **A.** Growth of CCRF-CEM cells was shown after transfection with miR-590 mimics or inhibitor or inactive control. The growth index as assessed at 0, 24, 48 and 72 h. **B.** Growth of Jurkat cells was shown after transfection with miR-590 mimics or inhibitor or inactive control. The growth index as assessed at 0, 24, 48 and 72 h. The growth index as assessed at 0, 24, 48 and 72 h. **C.** Cell cycle analysis of CCRF-CEM cells after treatment with miR-590 mimics, inhibitors or inactive control; **D.** Cell cycle analysis of Jurkat cells after treatment with miR-590 mimics, inhibitors or inactive control.

We explored the potential impact of miR-590 on T-ALL cell proliferation in CCRF-CEM and Jurkat cell lines. CCRF-CEM and Jurkat cells were transfected with miR-590 mimics or inhibitors or inactive control cel-mir-67. CCK-8 proliferation assay indicated that the cell proliferation is promoted in both of the miR-664-mimics-transfected T-ALL cell lines comparing with inactive control cel-mir-67-transfected cell lines (Figure 4A and 4B). Conversely, miR-590 inhibitors could inhibit the cell proliferation of CCRF-CEM and Jurkat cells (Figure 4A and 4B). The cell cycle analysis revealed that miR-664 mimics increase G1/S transition of CCRF-CEM and Jurkat cells, while miR-590 inhibitors inhibit it (Figure 4C and 4D). These results indicate that miR-590 promotes T-ALL cell proliferation by increasing G1/S transition.

### miR-590 promotes T-ALL cell migration and invasion

**Figure 5:**
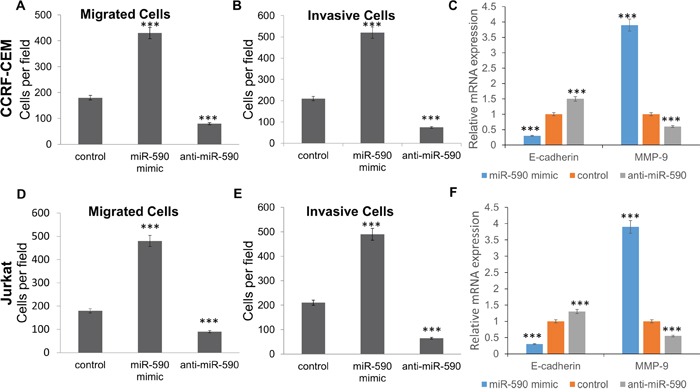
miR-590 regulates T-ALL cell invasion and migration **A.** Transwell analysis of CCRF-CEM cells migration after treatment with miR-590 mimics, inhibitors or inactive control; **B.** Transwell analysis of CCRF-CEM cells invasion after treatment with miR-590 mimics, inhibitors or inactive control; **C.** Expression of E-cadherin and MMP-9 in CCRF-CEM cells after treatment with miR-590 mimics, inhibitors or inactive control; **D.** Transwell analysis of Jurkat cells migration after treatment with miR-590 mimics, inhibitors or inactive control; **E.** Transwell analysis of Jurkat cells invasion after treatment with miR-590 mimics, inhibitors or inactive control; **F.** Expression of E-cadherin and MMP-9 in Jurkat cells after treatment with miR-590 mimics, inhibitors or inactive control. *p<0.05, **p<0.01, and ***p<0.001.

Interestingly, migration and invasion assay showed that miR-590 mimics promote the migration and invasion of CCRF-CEM and Jurkat cells comparing with the inactive cel-mir-67 control, whereas miR-590 inhibitors inhibit cell migration and invasion of the CCRF-CEM and Jurkat cells (Figure 5). Meanwhile, miR-590 mimics would decrease E-cadherin and increase MMP-9 expression in CCRF-CEM and Jurkat cells, while miR-590 inhibitors showed opposite effects (Figure 5C and 5F).

## DISCUSSION

In the past decades, miRNAs have be shown to be key transcriptional regulators in carcinogenesis [14]. Globally miRNA and gene expression profiles have provided valuable insights of molecular mechanisms for various cancers [15]. Thus we compared miRNA and gene expression data between T-ALL samples and healthy normal controls, and made further integrative bioinformatics analysis to identify potential miRNAs and corresponding target genes involved in carcinogenesis of T-ALL. The results revealed the potential role of miR-590 in T-ALL and the regulatory interaction between miR-590 and RB1. Further RT-PCR analysis in 395 patients with T-ALL and 316 age-matched healthy controls confirmed the up-regulation of miR-590 in T-ALL. Meanwhile, the expression of RB1 is highly correlated with miR-590 in T-ALL patients.

Increased expression of miR-590 has been found in hepatocellular carcinoma [16] and cervical cancer [17]. However, the relationship between miR-590 and T-ALL has never been reported before. Thus, our further study intended to investigate the biological functions of miR-590 in T-ALL. miR-590 mimics significantly promoted cell proliferation, migration and invasion in CCRF-CEM and Jurkat T-ALL cells. These results suggest that miR-590 plays a critical role in the carcinogenesis of T-ALL. Thereafter, we addressed the molecular mechanisms of miR-590 in promoting proliferation, migration and invasion of T-ALL cells. Our results further revealed that miR-590 promotes T-ALL cell proliferation by increasing G1/S transition. Western blots and luciferase assays revealed that RB1 is a target gene of miR-664. RB1 is a negative regulator of the cell cycle [13]. The deletion of RB1 was ever found in a T-ALL patient [18]. Down-regulation of RB1 is involved in various types of cancers, including osteosarcoma [19], Retinoblastoma [20] and lung adenocarcinoma [21]. miR-590 might take part in the carcinogenesis of T-ALL largely by negative regulating RB1. Moreover, miR-590 mimics would significantly decrease E-cadherin and increase MMP-9 expression in CCRF-CEM and Jurkat cells. E-cadherin is a calcium-dependent cell-cell adhesion molecule with pivotal roles in cell behavior [22]. Down-regulation of E-cadherin promotes cell invasion in various cancers, such as epithelial tumor [23] and breast cancer [24]. MMP-9 is the matrix metalloproteinase that would breakdown the extracellular matrix [25]. The increased MMP-9 is involved in cell migration and invasion in colorectal cancer [26] and acute leukemia [27]. The decreased E-cadherin and increased MMP-9 maybe part of the molecular mechanisms in miR-590 related T-ALL cell migration and invasion.

In conclusion, our results have shown that miR-590 promotes T-ALL cell proliferation, migration and invasion by directly targeting and down-regulating RB1, and further decrease E-cadherin and increase MMP-9. This novel miR-590/RB1 axis may provide new insights into the mechanisms underlying T-ALL carcinogenesis, and inhibition of miR-590 may be a potential therapeutic strategy for T-ALL in the future.

## MATERIALS AND METHODS

### miRNA and mRNA data

miRNA and mRNA profiles data of T-ALL and normal control samples were collected from GEO database (www.ncbi.nlm.nih.gov/gds, GSE56489, GSE45839, GSE56489, GSE41621, GSE46170, GSE67684). After quality control, 148 children with T-ALL and age-matched controls were included in miRNA analysis, while 250 children with T-ALL and age-matched controls were included in mRNA analysis.

### Identification of differentially expressed miRNA and mRNA pairs

The identification of differentially expressed miRNA and mRNA in children with T-ALL tissues were performed with Limma package on R platform using download miRNA and mRNA profiles data as mentioned above. The cutline of significantly differentially expressed miRNA and mRNA is P.value<0.01 (t test).

### miRNA target genes prediction

Human miRNA target genes prediction were performed with miRNA sequences that downloaded from the Rfam website (http://www.sanger.ac.uk/Software/Rfam) and satisfy the established criteria [8]. 3′ UTR sequences data for human genes were retrieved using EnsMart [9]. Repetitive elements in these sequences were masked by RepeatMasker [10] with repeat libraries for vertebrates, rodents, or primates, as appropriate. The target genes of miRNAs were predicted using miRanda [11] and TargetScan [12] methods. The predicted target genes supported by all the three methods were selected for further analysis.

### Integrative pathway analysis

The integrative pathway analysis of differentially expressed miRNAs and mRNAs was performed with Cytoscape software. The molecular network used in this analysis was constructed with predicted miRNA-mRNA interaction data and experimental validated human Protein-Protein interaction data (Downloaded from BioGrid database and HPRD database). The in-depth analysis of this pathway facilitated deciphering the complex interplay of differentially expressed miRNAs and corresponding target mRNAs and suggested their possible roles in the carcinogenesis of T-ALL. The pathway enrichment analysis is performed using fisher′ exact test.

### Ethics statement

The study was approved by the ethical committee of the Children's Hospital of Soochow University. Written informed consents were obtained from all the patients. The entire investigation conforms to the principles outlined in the Declaration of Helsinki.

### Patients and T-ALL samples

Patients undergoing T-ALL in the Children's Hospital of Soochow University were included. Blood samples from 395 patients with T-ALL and 316 age-matched healthy controls were collected between November 2013 and June 2015 at the Children's Hospital of Soochow University (Suzhou, China) and Affiliated Hospital of Jining Medical University (Jining, China), which had not received local or systemic treatment (Table 1).

### RNA extraction and RT-PCR

miRNA and mRNA expression levels were determined by using the qRT-PCR kit (Life Technologies, Beijing, China). Real-time PCR was performed in an QuantStudio^®^ 6 Flex Real-Time PCR System (Life Technologies, Beijing, China) by using a SYBR Green kit (TaKaRa, Tokyo, Japan), and the relative changes were quantified.

### Cell culture

The human T-ALL cell lines, CCRF-CEM was obtained from American Type Culture Collection (ATCC, USA). and Jurkat was obtained from the Chinese Center for Type Cultures Collections (CCTCC, China). The CCRF-CEM and Jurkat cell line was cultured in RPMI 1640 media (Life Technologies, Shanghai, China) and supplemented with 10% fetal bovine serum (FBS) (Life Technologies, Shanghai, China). Cells were maintained in a humidified atmosphere with 5% CO2 at 37°C.

### Cell transfection

CCRF-CEM and Jurkat cell lines were seeded in 24-well plates at 3×10^5^ cells/wells and incubated overnight. Transfection of the miR-590 miRNA mimic, the anti-miR-590, inactive control cel-mir-67 (Life Technologies, Shanghai, China), or pMIR-Report vectors was taken using Lipofectamine 2000 transfection reagent (Invitrogen, Shanghai, China) with 300 nmol of miRNA or 1μg/ml DNA plasmid, respectively. Total proteins of CCRF-CEM and Jurkat cells were isolated at 48 hours after transfection.

### Cell proliferation

Cell proliferations were measured using a Cell Counting Kit-8 (Dojindo, Kumamoto, Japan). CCRF-CEM and Jurkat T-ALL cells were plated in 24-well plates at 3×10^5^ cells/well. Then cells were incubated in 10% CCK-8 which was diluted in normal culture medium at 37°C for color conversion. Proliferation rates were determined at 24, 48 and 72 hours after transfection.

### Cell migration and invasion

Cell invasion and migration were measured using a transwell chamber (Corning, Shanghai, China) with and without Matrigel (Invitrogen, Shanghai, China). For the determination of CCRF-CEM and Jurkat T-ALL cells invasion, transwell chambers were placed into 24-well plates, and coated with 30 μl Matrigel, then incubated at 37°C for 40 minutes. In transwell assays with and without Matrigel, CCRF-CEM and Jurkat cells were trypsinized and then seeded in chambers at the density of 8×10^4^ cells/well at 48 hours after transfection. These cells were cultured in RPMI 1640 medium with 2% serum. Meanwhile 600 μl of 10% FBS-1640 was added to the lower chamber. After 24 hours, migrated CCRF-CEM and Jurkat cells were fixed in 100% methanol for 30 minutes. These non-migrated CCRF-CEM and Jurkat cells were removed by cotton swabs. After that cells on the bottom surface of the membrane were stained with the 0.1% crystal violet for 20 minutes. Images of CCRF-CEM and Jurkat cells were taken under a phase-contrast microscope.

### Luciferase assay

CCRF-CEM and Jurkat cells were seeded in 24-well plates at 1×10^5^ cells/well and incubated for 24 hours before transfection. In the reporter gene assay, the CCRF-CEM and Jurkat cells were co-transfected with 0.6 μg of pGL3-RB1-3′UTR or pGL3-RB1-3′UTR Mut plasmid, 0.06 ng of the phRL-SV40 control vector (Promega, Shanghai, China), and 100 nM miR-590 or control RNA using Lipofectamine 2000 (Invitrogen, Shanghai, China). The renilla and firefly luciferase activities were determined with a dual luciferase assay (Promega, Shanghai, China) 24 hours after transfection.

### Western blot

Proteins were separated by 12% SDS-PAGE gel and transferred onto nitrocellulose membranes (Bio-Rad, Shanghai, China). Membranes was blocked by 5% non-fat milk and incubated with anti-RB1 antibody (Abcam, Shanghai, China) or anti-β-actin antibody (Abcam, Shanghai, China). After being washed extensively, the secondary antibody (Abcam, Shanghai, China) was then added to the system. Finally, Immunoreactive protein bands were detected with the Enhanced Chemiluminescence (ECL) system.

### Statistical analysis

Experiments were repeated at least three times. Statistical analyses were performed with R. Values were expressed as means ± S.D. Differences between groups were determined with T-test. T-ALL analysis were considered to be significant when P. value<0.05.
